# COMMON PURPOSE: NETWORKING INITIATIVES TO FOSTER DEMENTIA-CAPABLE CARE FOR LIFELONG DISABILITIES

**DOI:** 10.1093/geroni/igz038.2755

**Published:** 2019-11-08

**Authors:** Catherine Taylor, Christine Gadbois, Sandra L Fournier, Phillip G Clark, Faith Helm

**Affiliations:** 1 Rhode Island Geriatric Education Center, University of Rhode Island, Kingston, Rhode Island, United States; 2 CareLink, Providence, Rhode Island, United States; 3 Neighborhood Health Plan of RI, Smithfield, Rhode Island, United States; 4 Rhode Island Geriatric Education Center, Kingston, Rhode Island, United States

## Abstract

As individuals with I/DD live longer, Alzheimer’s disease is on the rise, particularly in individuals with Down syndrome. Practitioners have recognized that the I/DD system does not possess the expertise to provide appropriate care for this population as it ages. A series of federally funded initiatives in Rhode Island – supported by the Centers for Medicare and Medicaid Services (CMS), the Administration for Community Living (ACL), and the Health Resources and Services Administration (HRSA) -- have begun to equip health care and direct care professionals to meet the needs of individuals with I/DD and AD. Using a “connector” model, the GWEP at RIGEC has woven together these efforts, aligned program goals from disparate funders, built connections between the Aging Network and the disability system, and worked with the National Task Group on Intellectual Disabilities and Dementia Practices (NTG) to create sustainable resources for the health care and direct care workforces.

